# Solving Cauchy reaction-diffusion equation by using Picard method

**DOI:** 10.1186/2193-1801-2-108

**Published:** 2013-03-13

**Authors:** Shadan Sadigh Behzadi

**Affiliations:** Department of Mathematics, Science and Research Branch, Islamic Azad University, Tehran, Iran

**Keywords:** Cauchy reaction-diffusion equation, Fuzzy number, Fuzzy-valued function, *h*-difference, Generalized differentiability, Picard method

## Abstract

In this paper, Picard method is proposed to solve the Cauchy reaction-diffusion equation with fuzzy initial condition under generalized *H*-differentiability. The existence and uniqueness of the solution and convergence of the proposed method are proved in details. Some examples are investigated to verify convergence results and to illustrate the efficiently of the method. Also, we obtain the switching points in examples.

## Introduction

As we know the fuzzy differential equations *FDE* are one of the important part of the fuzzy analysis theory that play major role in numerical analysis. For example, population models (Guo et al. 2003), the golden mean (Datta 2003), quantum optics and gravity (El Naschie 2005), control chaotic systems (Feng and Chen 2005; Jiang 2005), medicine (Abbod et al. 2001; Barro and Marin, 2002). Recently, some mathematicians have studied *FDE* (Abbasbandy and Allahviranloo 2000; Abbasbandy et al. 2004; Abbasbandy et al. 2005; Allahviranloo et al. 2007; Bede 2008; Bede and Gal 2005; Bede et al. 2007; Buckley and Feuring 2000; Buckley and Jowers 2006; Buckley et al. 2002; Chalco-Cano and Romn-Flores 2006; Chalco-Cano and Romn-Flores et al. 2007; Chapko and Johansson 2012; Chen and Ho 1999; Cho and Lan 2007; Congxin and Shiji 1993; Diamond 1999; Diamond 2002; Ding et al. 1997; Dubois 1982; Dou and Hon 2012; Fard 2009a; Fard and Bidgoli b; Fard and Bidgoli 2010; Fard and Kamyad 2011; Fard et al. 2009; Fei 2007; Jang et al. 2000; Jowers et al. 2007; Kaleva 1987, 1990, 2006; Lopez 2008; Ma et al. 1999; Mizukoshi et al. 2007; Oberguggenberger and Pittschmann 1999; Papaschinopoulos 2007; Puri and Ralescu 1983; Seikkala 1987; Solaymani Fard and Ghal-Eh 2011; Song et al. 2000). The fuzzy partial differential equations *FPDE* are very important in mathematical models of physical, chemical, biological, economics and other fields. Some mathematicians have studied solution of *FPDE* by numerical methods (Afshar Kermani and Saburi 2007; Allahviranloo 2002; Barkhordari Ahmadi and Kiani 2011; Buckley and Feuring 1999; Chen et al. 2009; Farajzadeh et al. 2010; Moghadam and Jalal 2011; Rouhparvar et al. 2010; Verma et al. 2009). In this work, we present the Picard method to solve the Cauchy reaction-diffusion equation as follows: 1

With fuzzy initial condition: 2

The structure of this paper is organized as follows: In section “Basic concepts”, some basic notations and definitions in fuzzy calculus are brought. In section “Description of the method”, Eqs.(1,2) are solved by Picard method. The existence and uniqueness of the solution and convergence of the proposed method are proved in section “Existence and convergence analysis” respectively. Finally, in section “Numerical examples”, the accuracy of method by solving some numerical examples are illustrated and a brief conclusion is given in section “Conclusion”.

**Figure 1:**
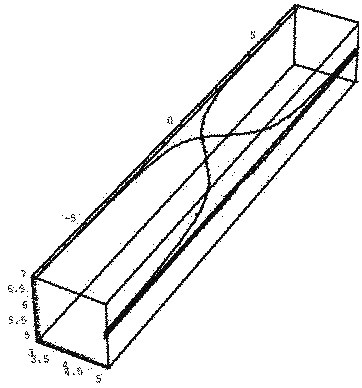
**The results of Example 5.1 for (****(*x*, 0.6, 0.1),****(x, 0.6, 0.1)).**

## Basic concepts

Here basic definitions of a fuzzy number are given as follows, (Allahviramloo 2005; Dubois and Prade 2005; Kauffman and Gupta 1991;Nguyen 1978;Zadeh 1965)

### Definition 2.1

An arbitrary fuzzy number in the parametric form is represented by an ordered pair of functions which satisfy the following requirements:

(i) is a bounded left-continuous non-decreasing function over [0,1],
(ii) is a bounded left-continuous non-increasing function over [0,1],
(iii)

### Definition 2.2

For arbitrary fuzzy numbers, we use the distance (Hausdorff metric) (Goetschel and Voxman 1986) and it is shown (Puri and Ralescu 1986) that (*E*^**1**^, D) is a complete metric space and the following properties are well known: 

### Definition 2.3

Consider,. If there exists such that then is called the *H*- difference of and, and is denoted by (Bede and Gal 2005).

### Proposition 1

If is a continuous fuzzy-valued function then is differentiable, with derivative (Bede and Gal 2005).

### Definition 2.4

(see (Bede and Gal 2005)) Let and *x*_0_ ∈ (*a*, *b*). We say that is generalized differentiable at *x*_0_ (Bede-Gal differentiability), if there exists an element, such that:

i) for all *h* > 0 sufficiently small, ∃ *f*(*x*_0_ + *h*) ⊖ *f*(*x*_0_), ∃ *f*(*x*_0_) ⊖ *f*(*x*_0_ − *h*) and the following limits hold:
  or
ii) for all *h* >V0 sufficiently small, ∃ *f*(*x*_0_) ⊖ *f*(*x*_0_ + *h*), ∃ *f*(*x*_0_ − *h*) ⊖ *f*(*x*_0_) and the following limits hold:
  or
iii) for all *h*>0 sufficiently small, ∃ *f*(*x*_0_ + *h*) ⊖ *f*(*x*_0_), ∃ *f*(*x*_0_ − *h*) ⊖ *f*(*x*_0_) and the following limits hold:
  or
iv) for all *h* > 0 sufficiently small, ∃ *f*(*x*_0_) ⊖ *f*(*x*_0_ + *h*), ∃ *f*(*x*_0_) ⊖ *f*(*x*_0_ − *h*) and the following limits hold:

### Definition 2.5

Let. We say is (i)-differentiable on (*a*, *b*) if is differentiable in the sense (i) of Definition (2.7) and similarly for (ii), (iii) and (iv) differentiability.

### Definition 2.6

(see (Chalco-Cano and Romn-Flores 2006)) Let and *x*_0_ ∈ (*a*, *b*). A point *x*_0_ ∈ (*a*, *b*) is said to be a switching point for the differentiability of, if in any neighborhood *V* of *x*_0_ there exist points *x*_1_ < *x*_0_ < *x*_2_ such that

(type 1) is differentiable at *x*_1_ in the sense (*i*) of Definition (2.6) while it is not differentiable in the sense (*ii*) of Definition (2.6), and is differentiable at *x*_2_ in the sense (*ii*) of Definition (2.6) while it is not differentiable in the sense (*i*) or Definition (2.6), or

(type 2) is differentiable at *x*_1_ in the sense (*ii*) of Definition (2.6) while it is not differentiable in the sense (*i*) of Definition (2.6), and is differentiable at *x*_2_ in the sense (*i*) of Definition (2.6) while it is not differentiable in the sense (*ii*) or Definition (2.6).

### Proposition 2

(see (Chalco-Cano and Romn-Flores 2006)) Let and *x*_0_ ∈ (*a*, *b*).

- If *x*_0_ ∈ (*a*, *b*) is a switching point for the differentiability of of type 1, then is differentiable at *x*_0_ in the form (*iv*).
- If *x*_0_ ∈ (*a*, *b*) is a switching point for the differentiability of of type 2, then is differentiable at *x*_0_ in the form (*iii*).

### Definition 2.7

A triangular fuzzy number is defined as a fuzzy set in *E*^1^, that is specified by an ordered triple *u* = (*a*, *b*, *c*) ∈ *R*^3^ with *a* ≤ *b* ≤ *c* such that are the endpoints of *r*-level sets for all *r* ∈ [0, 1], where and. Here,, which is denoted by *u*(1). The set of triangular fuzzy numbers will be denoted by *E*^1^.

### Definition 2.8

(see (Chalco-Cano and Romn-Flores 2006)) The mapping for some interval *T* is called a fuzzy process. Therefore, its *r*-level set can be written as follows: 

### Definition 2.9

(see (Chalco-Cano and Romn-Flores 2006)) Let be Hukuhara differentiable and denote. Then, the boundary function and are differentiable (or Seikkala differentiable) and 

If *f* is (*ii*)-differentiable then 

## Description of the method

To obtain the approximation solution of Eqs.(1,2), based on Definition (2.6) we have two cases as follows:

Case (1): is (*i*)-differentiable, in this case we have, 3

Case (2): is (*ii*)-differentiable, in this case we have, 4

Now, we can write successive iterations (by using Picard method) as follows:

Case (1): 5

Case (2): 6

### Remark 1

For we have cases as follows:

Case (1): and be (i)-differentiable and and be (ii)-differentiable 

Case (2): is (*i*)-differentiable and is (ii)-differentiable and is (*ii*)-differentiable and is (i)-differentiable 

### Remark 2

We discuss about switching points as follows:

Case (1): is (*i*)-differentiable

If and and *r* ∈ [0, 1] then we have and *x*_0_ is a switching point in the form (*iv*).

If and and *r* ∈ [0, 1] then we have and *x*_1_ is a switching point in the form (*iv*).

Case (2): is (*ii*)-differentiable

If and and *r* ∈ [0, 1] then we have and *x*_0_ is a switching point in the form (*iii*).

If and and *r* ∈ [0, 1] then we have and *x*_1_ is a switching point in the form (*iii*).

Case (3): is (*i*)-differentiable

If then we have.

If and and *r* ∈ [0, 1] then we have and *x*_0_ is a switching point in the form (*iv*).

Case (4): is (*ii*)-differentiable

If then we have.

If and and *r* ∈ [0, 1] then we have and *x*_1_ is a switching point in the form (*iii*).

## Existence and convergence analysis

In this section we are going to prove the existence and uniqueness of the solution and convergence of the method by using the following assumptions.

Consideris bounded for all *x* ∈ [*a*, *b*] and 

Let, 

### Lemma 1

If and *λ* ∈ *R*, then,

(i)
(ii)

**Proof (i).** By the definition of *D*, we have, 

**Proof (ii):**

### Theorem 1

Let 0 < *α* < 1, then Eqs.(1,2), have an unique solution and the solution obtained from the relation (8) using Picard method converges to the exact solution of the problems (1, 2) when is (ii)-differentiable.

**Proof.** Let and be two different solutions of Eqs.(1, 2) then 

From which we get. Since 0 < *α* < 1, then. Implies.

Also, we have 

Since, 0 < *α* < 1, then as *n* → *∞*. Therefore,. □

### Remark 3

The proof of other case is similar to the previous theorems.

## Numerical examples

In this section, we solve the Cauchy reaction-diffusion equation by using the Picard method. The program has been provided with Mathematica 6 according to the following algorithm where *ε* is a given positive value.

**Algorithm :**

### Example 5.1

Consider the Cauchy reaction-diffusion equation as follows: 7

With initial condition: 8

*ϵ* = 10^−4^. *x* = 0 is a switching point.

Case (1): *n* = 22 and *α* = 0.8652.

Case (2): *α* = 0.84569.

Table 1 shows that, the approximation solution of the Cauchy reaction-diffusion equation is convergent with 25 iterations by using the Picard method when is (ii)-differentiable.

**Table 1:** **Numerical results for Example 5.1**

***x***		
−0.2	0.34245	0.68532
−0.1	0.35571	0.67259
0.0	0.36438	0.66135
0.1	0.35724	0.67056
0.2	0.34862	0.67843
0.3	0.33597	0.68453
0.4	0.32551	0.69064

### Example 5.2

Consider the Cauchy reaction-diffusion equation as follows: 9

With initial condition: 10

*ϵ* = 10^**−3**^.

Case (1): *α* = 0.7546.

Table 2 shows that, the approximation solution of the Cauchy reaction-diffusion equation is convergent with 17 iterations by using the Picard method when is (i)-differentiable.

**Table 2:** **Numerical results for Example** 5.2

***x***		
0.1	0.3726508	0.7023467
0.2	0.3827526	0.6939411
0.3	0.3957162	0.6848745
0.4	0.4136805	0.6612719
0.5	0.4372286	0.6559328
0.6	0.4438574	0.6337306

Case (2): *α* = 0.7762.

Table 3 shows that, the approximation solution of the Cauchy reaction-diffusion equation is convergent with 21 iterations by using the Picard method when is (ii)-differentiable.

**Table 3:** **Numerical results for Example** 5.2

***x***		
0.1	0.4646316	0.7954225
0.2	0.4717726	0.7844835
0.3	0.4823549	0.7737607
0.4	0.5074658	0.7528153
0.5	0.5263437	0.7474326
0.6	0.5309183	0.7215178

## Conclusion

The Picard method has been shown to solve effectively, easily and accurately a large class of nonlinear problems with the approximations which convergent are rapidly to exact solutions. In this work, the Picard method has been successfully employed to obtain the approximate solution of the Cauchy reaction-diffusion equation under generalized *H*-differentiability.

## References

[CR1] Abbasbandy S, Allahviranloo T (2000). Numerical solutions of fuzzy differential equations by Taylor method. J Comput Meth Appl Math.

[CR2] Abbasbandy S, Allahviranloo T, Lopez-Pouso O, Nieto J. J. (2004). Numerical methods for fuzzy differential inclusions. Comput Math Appl.

[CR3] Abbasbandy S, Nieto J. J., Alavi M (2005). Tuning of reachable set in one dimensional fuzzy differential inclusions. Chaos Soliton Fractals.

[CR4] Abbod MF, Von Keyserlingk DG, Linkens DA, Mahfouf M (2001). Survey of utilisation of fuzzy technology in medicine and healthcare. Fuzzy Sets Syst.

[CR5] Afshar Kermani M, Saburi F (2007). Numerical method for fuzzy partial differential equations. Appl Math Sci.

[CR6] Allahviranloo T (2002). Difference methods for fuzzy partial differential equations. Comput Methods Appl Math.

[CR7] Allahviramloo T (2005). The Adomian decomposition method for fuzzy system of linear equations. Appl Mathematics and Comput.

[CR8] Allahviranloo T, Ahmady N, Ahmady E (2007). Numerical solution of fuzzy differential equations by predictorŰcorrector method. Inform Sci.

[CR9] Barkhordari Ahmadi M, Kiani NA (2011). Solving fuzzy partial differential equation by differential transformation method. J Appl Math.

[CR10] Barro S, Marin R (2002). Fuzzy logic in medicine.

[CR11] Bede B (2008). Note on Numerical solutions of fuzzy differential equations by predictor-corrector method. Inform Sci.

[CR12] Bede B, Gal SG (2005). Generalizations of the differentiability of fuzzy number valued functions with applications to fuzzy differential equation. Fuzzy Set Syst.

[CR13] Bede B, Imre J, Rudas C, Attila L (2007). First order linear fuzzy differential equations under generalized differentiability. Inform Sci.

[CR14] Buckley JJ, Feuring T (1999). Introduction to fuzzy partial diferential equations. Fuzzy Sets and Syst.

[CR15] Buckley, JJ (2000). Fuzzy differential equations. Fuzzy Set Syst.

[CR16] Buckley JJ, Jowers LJ (2006). Simulating Continuous Fuzzy Systems.

[CR17] Buckley JJ, Feuring T, Hayashi Y (2002). Linear systems of first order ordinary differential equations: fuzzy initial conditions. Soft Comput.

[CR18] Chalco-Cano Y, Román-Flores H (2006). On new solutions of fuzzy differential equations. Chaos Soliton Fractals.

[CR19] Chalco-Cano Y, Román-Flores H, Rojas-Medar MA, Saavedra O, Jimnez-Gamero M (2007). The extension principle and a decomposition of fuzzy sets. Inform Sci.

[CR20] Chalco-Cano Y, Roman-Flores H, Jimnez-Gamero MD (2011). Generalized derivative and π-derivative for set-valued functions. Inf Sci.

[CR21] Chapko R, Johansson BT (2012). On the numerical solution of a Cauchy problem for the Laplace equation via a direct integral equation approach. Inverse Problems and Imaging.

[CR22] Chen CK, Ho SH (1999). Solving partial differential equations by two-dimensional differential transform method. Appl Math Comput.

[CR23] Chen Y-Y, Chang Y-T, Chen B-S (2009). Fuzzy solutions to partial differential equations: adaptive approach. IEEE Trans on Fuzzy Syst.

[CR24] Cho YJ, Lan HY (2007). The existence of solutions for the nonlinear first order fuzzy differential equations with discontinuous conditions. Dyn Contin Discrete.

[CR25] Congxin W, Shiji S (1993). Exitance theorem to the Cauchy problem of fuzzy differential equations under compactance-type conditions. Inform Sci.

[CR26] Datta DP (2003). The golden mean, scale free extension of real number system, fuzzy sets and 1/f spectrum in physics and biology. Chaos, Solitons and Fractals.

[CR27] Diamond P (1999). Time-dependent differential inclusions, cocycle attractors and fuzzy differential equations. IEEE Trans Fuzzy Syst.

[CR28] Diamond, P (2002). Brief note on the variation of constants formula for fuzzy differential equations. Fuzzy Set Syst.

[CR29] Ding Z, Ma M, Kandel A (1997). Existence of solutions of fuzzy differential equations with parameters. Inform Sci.

[CR30] Dubois D, Prade H (1980). Theory and application, fuzzy sets and systems.

[CR31] Prade H, Dubois, D (1982). Towards fuzzy differential calculus: Part 3, differentiation. Fuzzy Set Syst.

[CR32] Dou FF, Hon YC (2012). Kernel-based approximation for Cauchy problem of the time-fractional diffusion equation.

[CR33] El Naschie MS (2005). From experimental quantum optics to quantum gavity via a fuzzy Kahler manifold. Chaos, Solitons and Fractals.

[CR34] Farajzadeh A, Hossein Pour A, Amini M (2010). An Explicit Method for Solving Fuzzy Partial Differential Equation. Int Math Forum.

[CR35] Fard OS (2009). A numerical scheme for fuzzy cauchy problems. J Uncertain Syst.

[CR36] Fard O. S. (2009). An iterative scheme for the solution of generalized system of linear fuzzy differential equations. World Appl Sci J.

[CR37] Fard OS, Kamyad AV (2011). Modified k-step method for solving fuzzy initial value problems. Iran J Fuzzy Syst.

[CR38] Fard OS, Hadi Z, Ghal-Eh N, Borzabadi AH (2009). A note on iterative method for solving fuzzy initial value problems. J Adv Res Sci Comput.

[CR39] Fard OS, Bidgoli TA, Borzabadi AH (2010). Approximate-analytical approach to nonlinear FDEs under generalized differentiability. J Adv Res Dyn Control Syst.

[CR40] Fei W (2007). Existence and uniqueness of solution for fuzzy random differential equations with non-Lipschitz coefficients. Inform Sci.

[CR41] Feng G, Chen G (2005). Adaptative control of discrete-time chaotic system: a fuzzy control approach. Chaos, Solitons and Fractals.

[CR42] Goetschel R, Voxman W (1986). Elementary calculus. Fuzzy Sets Syst.

[CR43] Guo M, Xue X, Li R (2003). Impulsive functional differential inclusions and fuzzy population models. Fuzzy Sets Syst.

[CR44] Jang MJ, Chen CL, Liy YC (2000). On solving the initial-value problems using the differential transformation method. Appl Math Comput.

[CR45] Jiang W, Guo-Dong Q, Bin D (2005). H∞ variable univerese adaptative fuzzy control for chaotic systems. Chaos, Solitons and Fractals.

[CR46] Jowers LJ, Buckley JJ, Reilly KD (2007). Simulating continuous fuzzy systems. Inform Sci.

[CR47] Kaleva O (1987). Fuzzy differential equations. Fuzzy Set Syst.

[CR48] Kaleva, O (1990). The Cauchy problem for fuzzy differential equations. Fuzzy Set Syst.

[CR49] Kaleva, O (2006). A note on fuzzy differential equations. Nonlinear Anal.

[CR50] Kauffman A, Gupta MM (1991). Introduction to Fuzzy Arithmetic:Theory and Application.

[CR51] Lopez RR (2008). Comparison results for fuzzy differential equations. Inform Sci.

[CR52] Ma M, Friedman M, Kandel A (1999). Numerical solutions of fuzzy differential equations. Fuzzy Set Syst.

[CR53] Mizukoshi MT, Barros LC, Chalco-Cano Y, Román-Flores H, Bassanezi RC (2007). Fuzzy differential equations and the extension principle. Inform Sci.

[CR54] Moghadam MM, Jalal I (2011). Finite Volume Methods for Fuzzy Parabolic Equations. J Math Comput Sci.

[CR55] Nguyen HT (1978). A note on the extension principle for fuzzy sets. J Math Anal Appl.

[CR56] Oberguggenberger M, Pittschmann S (1999). Differential equations with fuzzy parameters. Math Mod Syst.

[CR57] Papaschinopoulos G, Stefanidou G, Efraimidi P (2007). Existence uniqueness and asymptotic behavior of the solutions of a fuzzy differential equation with piecewise constant argument. Inform Sci.

[CR58] Puri ML, Ralescu DA (1983). Differentials of fuzzy functions. J Math Anal Appl.

[CR59] Puri ML, Ralescu D (1986). Fuzzy random variables. J Math Anal Appl.

[CR60] Seikkala S (1987). On the fuzzy initial value problem. Fuzzy Set Syst.

[CR61] Solaymani Fard O, Ghal-Eh N (2011). Numerical solutions for linear system of first-order fuzzy differential equations with fuzzy constant coefficients. Infor Sci.

[CR62] Song S, Guo L, Feng C (2000). Global existence of solutions to fuzzy differential equations. Fuzzy Set Syst.

[CR63] Rouhparvar H, Abbasbandy S, Allahviranloo T (2010). Existence and uniqueness of solution of an uncertain characteristic cauchy reaction-diffusion equation by Adomian decomposition method. Math Comput Appl.

[CR64] Verma P, Sing P, George KV, Singh HV, Devotta S, Singh RN (2009). Uncertainty analysis of transport of water and pesticide in an unsaturated layered soil profile using fuzzy set theory. Appl Math Modelling.

[CR65] Zadeh LA (1965). Fuzzy Sets. Inf and Control.

[CR66] (1991). Fuzzy sets theory and its applications.

